# New approach for localization and smart data transmission inside underground mine environment

**DOI:** 10.1007/s42452-021-04589-2

**Published:** 2021-05-01

**Authors:** Ankita RayChowdhury, Ankita Pramanik, Gopal Chandra Roy

**Affiliations:** 1 Department of Electronics and Telecommunication Engineering, Indian Institute of Engineering, Science & Technology, Shibpur, West Bengal India 711103; 2 Department of Mining Engineering, Indian Institute of Engineering, Science & Technology, Shibpur, West Bengal India 711103

**Keywords:** Underground mine, Visible light communication, Location tracking, Long range technology, SNR, Received signal strength

## Abstract

This paper presents an approach to access real time data from underground mine. Two advance technologies are presented that can improve the adverse environmental effect of underground mine. Visible light communication (VLC) technology is incorporated to estimate the location of miners inside the mine. The distribution of signal to noise ratio (SNR) for VLC system is also studied. In the second part of the paper, long range (LoRa) technology is introduced for transmitting underground information to above the surface control room. This paper also includes details of the LoRa technology, and presents comparison of ranges with existing above the surface technologies.

## Introduction

Underground mining industry is essential to run human civilization, thus demanding technological advancement in its infrastructure and work environment [1]. Presence of different toxic and combustible gases make mine environment vulnerable for the miners working inside. Hazardous work environment can even become deadly for the workers [2].

Underground mine environment is very different from the surface environment due to the presence of poisonous substances, toxic gases, corrosive water and dusts [3]. Moreover mine structure is full of twists and turns with long-walls. Excavation process makes a mine structure dynamic. Due to mining operations, accidents like explosion, roof fall (common in underground mines), and landslides (common in opencast) are very frequent in occurrence in a mine thus endangering life of miners. For faster response to any untoward event, proper communication connectivity inside the mine, among the miners and also with above the surface personnel is very important. Safety measures like proper exchange of information regarding the environmental conditions, and transmission of position-based information of the miners during emergency [4, 5], should be considered. This will also help in improvement of miner’s mental and physical health. Implementation of such technologies inside a mine is a challenging task due to the perilous and adverse mine environment.

Various mine communication system, namely wired communication systems (coaxial, fiber optics, twisted pair), radio communication system, carrier current communication system and a mix of these two named as hybrid system has been developed over the years. Among these, wired communication is most inappropriate; as mine structure is dynamic and underlying wire get damaged during mining operations and can cause accidents inside the mine [6]. Radio communication system provides wireless connectivity but uses RF band for establishing communication. RF signal experiences multipath fading and path-loss while propagating inside the mine [6]. Moreover, it is not feasible to use surface technologies in underground mine. Underground mine is global positioning system (GPS) denied area, as signal gets attenuated easily in the presence of heavy mining machineries. Also GPS terminals are unable to process the beacons received from the satellite because of the obstructions present in underground mine [7]. Other wireless technologies such as radio frequency identification (RFID), wireless fidelity (Wi-Fi) and Zigbee, popular for indoor positioning above the surface [8] also fail to provide expected outcome in underground mine because of signal attenuation and path-loss, as these technologies operate in RF band.

Mining operation emits different hazardous and combustible gases, like methane (CH4) and carbon mono-oxide (CO). Thus the use of high energy transmission must be avoided as it might lead to a lethal accident. So the transmitted energy should not exceed 25 mJ inside a coal mine. Thus it is essential to find a technology that requires low power for transmission.

In view of these challenges new emerging and promising technology, suitable for mine environment has been adopted. Visible light communication (VLC) technology is introduced in mine area to gather location information as it offers illumination, high speed data communication and localization [9]. It offers low cost, precise and accurate localization information [10, 11]. Unlike conventional RF- based system, VLC system uses unlicensed and unregulated [11] visible light spectrum (with wavelength 380–740 nm and frequency from 400 to 790 THz). VLC system is capable of providing high data rate. The signal is immune to electromagnetic interference. Line-of-sight (LOS) communication ensures reliability and security. VLC system is less prone to attenuation due to the use of high frequency. In this system LEDs act as transmitters and receivers comprises of photo-diodes (PDs) or image sensors (ISs). LEDs also help to illuminate [11] the gloomy mine environment which helps to boost the mental condition of miners. Along with this, low power wide area network (LPWAN) technology is also integrated in mine [12] for data transmission.

Considering the dynamic nature of mine, the adopted technology should be easy to install and should offer long range for reliable data transmission. Long range (LoRa) technology, discussed in this paper, comes under LPWAN technology and works best for underground scenario [12]. Characteristics like: use of unlicensed frequency band [13], unique characteristics of physical layer [14], − 150 dBm receiver sensitivity [15]; makes LoRa technology most suitable for implementation in underground mine.

VLC receiver will transmit the received information to above the surface personnel for running the algorithm in order to track miner’s movement inside the mine. LoRa technology helps to transmit the data. Because of its low power consumption feature, this technology can easily be installed inside the mine without interrupting the normal mining operation.

This paper presents the key features for location tracking by VLC system and also discusses the possibility of seamless data transmission technology from underground to above the ground. To the best knowledge of the authors, use of such technology to enable IoT inside a mine has not been done yet. To reduce intercellular interference, a novel zone division technique is also proposed in the work.

The main contributions of this paper are as follows:

- This work discusses about different parameters that need to be considered for implementing VLC system in underground mine.
- This work gives brief description about the LoRa technology and discusses about the useful features of this technology.
- Location estimation algorithm is proposed and simulation result of SNR performance of VLC system is studied.
- Experimental result for range test of LoRa technology is presented along with comparison with other existing technologies.

Rest of the paper is organised as follows: Sect. 2 discusses about technological advancement in underground mine, Sect. 3 presents state of the art of these technologies, Sect. 4 proposes system model adopted in this work and Sect. 5 gives the results and discussions. Finally paper is concluded in Sect. 6.

## Technological advancement in underground mine

In underground mining industry advance communication networking can be achieved by integrating new technologies. Better communication ensures safe working environment and increases productivity. In this section, two potential technologies that can be incorporated in underground mine are discussed.

### Wireless connectivity using LoRa technology

LoRa was introduced by Semtech in 2012 [14]. Other recent technologies for IoT environment are; wireless-fidelity (Wi-Fi) and Bluetooth 5.0. The superiority of LoRa technology in comparison with other IoT technologies is presented in Table 1.

**Table 1:** Comparison between Wi-Fi, Bluetooth and LoRa Technology for IoT

Specifications	Bluetooth 5.0	Wi-Fi	LoRa
Modulation	Frequency hopping spread spectrum	Frequency hopping spread spectrum	Chirp spread spectrum
Frequency	2.4 GHz	2.4 GHz and 5 GHz	EU (433 MHz, 868 MHz), US (915 MHz), Asia (430 MHz)
Range	10–100 m	100 m	10 km (approx.)
Data rate	1, 2, 3 Mbps	0.1–54 Mbps	30–50 Kbps
Power usage	Medium	High	Low
Application	Use to connect laptop, PDAs, phones, headsets within vicinity	Wireless LAN connectivity, broadband internet access	Use to connect laptop, PDAs, phones, headsets, sensors and other any data communication device

Specifications of LoRa technology is given in Table 2. It modulates the signal with chirp spread spectrum (CSS) technique [15]. Key characteristics of this modulation scheme are: (i) it uses more bandwidth as narrow band signal is spread over wide band while transmission, (ii) it increases link budget, (iii) provides better immunity to channel noise, (iv) Doppler effects and multipath fading. These unique features of LoRa technology, makes it suitable for underground mine environment.

**Table 2:** Technical specifications of LoRa technology [16]

Specifications	LoRa
Modulation	Chirp spread spectrum
Band	Sub-GHz ISM-EU (433 MHz, 868 MHz), US (915 MHz), Asia (430 MHz)
Data Rate	0.3–37.5kbps (LoRa) and 50kbps (FSK)
Range	5 km (Urban) and 15 km (Rural)
MAC	Unslotted ALOHA
Topology	Star of stars
Payload length	Up to 250B (depends on spreading factors and region)
Handover	End devices do not join single base station
Link budget	154 dB
Authentication and Encryption	AES 128b
Scalability	Yes
Mobility/Localization	Yes
Power efficiency	Very high

LoRa technology is defined by two layers: (1) LoRaPHY (LoRa physical layer) and (2) LoRaWAN (MAC protocol layer). To establish communication, network is framed using multiple end nodes (sensors), deployed in the mine, maintaining star-of-star topology, and connected with single/multiple gateways.

#### LoRaPHY

LoRa physical layer is described in LoRaPHY. It is a private network, which describes LoRa modulation scheme, bandwidth, spreading factor and code rate. Adopted CSS modulation scheme uses different frequency chirps which vary linearly over time while encoding [17]. Interference and multipath fading can be reduced by using spreading factor ranging from 7 to 12. Spreading factor helps to increase the communication range. Each chirp encodes one symbol of information. Adoptive transmission parameters and flexible coverage range of LoRa technology is governed by this spreading factor. This technology also offers different bandwidth; like 125 kHz, 250 kHz, or 500 kHz [18] depending on the application. Table 3 describes how LoRa transmission characteristics are controlled by different SF for 125 kHz.

**Table 3:** LoRa spreading factors for 125 kHz bandwidth [19]

Spreading factor	Symbols/second	SNR limit	Bit rate
7	976	− 7.5	5469
8	488	− 10	3125
9	244	− 12.5	1758
10	122	− 15	977
11	61	− 17.5	537
12	30	− 20	293

Adaptive data rate (ADR) mechanism allows the data rate to be increased for the end nodes near to the gateway. Data rate can vary from 300 bps to 37.5 Kbps.

#### LoRaWAN

Protocols of this technology is defined by LoRaWAN. IBM, Actility, Semtech and Microchip [20] developed LoRa Alliance which framed different LoRaWAN protocols. These protocols offer long range, low power and low data rate to the end devices to establish communication. End devices (sensors), gateways, and network servers; are three different components that sum up LoRaWAN. Star-of star topology is formed by the end devices and gateways in the network. At the back end, network server stores all the information gathered from the field. End device that communicates with the gateway are of three types:

- Class A: used for uplink communication, consumes less power and rigid to downlink communication.
- Class B: suitable for both uplink and downlink packet transmission.
- Class C: Ideal devices for packet transmission, also consumes maximum power.

LoRa technology uses unlicensed band of frequencies. Table 4 gives a list of operating frequency used across the world.

**Table 4:** Operating frequency of different country [21]

Country	Operating frequency band (MHz)
Europe	863–870
North America	902–928
China	470–510
Korea	920–925
Japan	920–925
European Union	433
Australia	915–928
India	865–867

Security of the technology is governed by IEEE 802.15.4 [21]. Two session keys, network session key and application session key, are used to ensure security. To further increase security during transmission, another 128-bit AES key can be introduced at each node.

### Different approaches of location estimation using VLC system

LEDs in VLC system act as transmitter and receiver units consist of PDs or ISs. Implementing VLC system inside the mine is mainly because of use of LEDs. It is feasible to modulate LED light intensity with high frequency such as 300 MHz because of its high switching speed capability. Also LEDs can transmit data without causing visible flickers [22, 23]. LEDs offer long life, low power consumption, robustness, reliability, security, and illumination. The receiver unit can be PDs [24] or ISs [25]. For both the cases, distance between transmitter and receiver is calculated by direct or indirect method based on power received [26]. PDs are cheaper, offer high data rate and are more energy efficient. On the other hand ISs are costly and offer low data rate. To establish communication between transmitter and receiver in VLC system, LOS is required.

Over many years researchers have developed various algorithms for location calculation using VLC system. In general, there are two different approaches:

(a) Direct positioning: In this method, received signal is directly considered for extracting location related information; no other parameters are considered or measured in advance for calculation [27]. This method gives less error in calculation and provides optima solution.

(b) Two-step Positioning: In this method, different parameters are measured, assumptions are made, and design constraints are considered prior to calculation of location. The received signal is recorded and then with all these parameters location estimation is done [28, 29]. Since, it involves more calculation, this method requires high data storage and communication facility, but it is less complex and provides suboptimal solution.

Table 5 illustrate different techniques adopted in literatures for acquiring position related information using VLC system.

**Table 5:** Different techniques used in VLC system

Technique	Characteristics
Received signal strength (RSS) [30, 31]	Very common and useful, low cost solution
Time of arrival (TOA) [32]/Time difference of arrival (TDOA) [33]	Synchronization is needed for this. More complex than RSS
Angle of arrival (AOA) [34, 35]	Most efficient technique and low cost. This parameter is measured based on received power levels at receiver
Proximity based method [36, 37]	Not very efficient. It is used in application where very accurate position information is not required
Geometric methods [33]	Triangulation, trilateration methods comes under this category
Statistical distribution method [38]	Exploits statistical properties of the measurements is used
Finger printing method [39, 40]	Two methods are opted, online and offline. The final calculation is done by comparing the result of two processes
Optimal power allocation approach	It is designed at the transmitter end. LEDs power level is set to optimum level and power and illumination of the LEDs are considered

## State of the art

This section consists of two subsections. The first subsection elaborates how LoRa technology has been proven to be the best in different challenging environment. In the second subsection, literature survey of VLC system for location estimation is presented.

### About wireless communication inside underground mine

The main challenge in establishing communication setup in underground mine is its dynamic and adverse nature. Since RF signal cannot propagate without remarkable attenuation, above the surface technologies; like wireless local area network (WLAN) or wireless personal area network (WPAN) failed to provide desirable result in underground mine. In work [6], authors described different frequencies that may be used in underground mine communication.

In [41] LoRa technology was deployed in both indoor and outdoor environment. For outdoor environment, glass buildings, sandstone buildings of up to seven stories, terrain of flat river plain and 50 m high hilly area of Glasgow city was selected. Transmitted signal strength was good in the outdoor environment but in indoor, an enclosed stairwell was considered, where signal strength was nullified. Authors of the paper suggested using different gateways for data transmission in two different environments.

Authors in [42] applied LoRa technology for sailing monitoring system. Brazil Olympics sailing venue was chosen to conduct the test. Frequency of 433 MHz and 250 kHz and 500 kHz bandwidth were selected for the operation. Obtained results confirmed that data rate and coverage range of the technology was controlled by spreading factor and bandwidth. Maximum of 2 km coverage range was obtained with spreading factor 7, 9 and 12.

Low cost fine-grained air monitoring system was developed using LoRaWAN technology in [43]. In a campus, sensors were densely deployed and LoRaWAN technology was adopted to collect the sensor data. The test confirmed reliable packet delivery capability of the technology with very less power consumption.

A smart phone application was developed by the authors in [44] christened LoRa-Wizard. Purpose of the test was to characterize LoRa for in-soil propagation. Different LoRa characteristics, like high flexibility, high reliability, low cost and ease of use were studied in the test.

LoRa technology is recently being used in many such applications for collecting sensor data and for reliable data transmission, work in [45]. Here LoRa technology was used to collect temperature, humidity, and luminosity and CO_2_ sensor data from the city of Bologna. To the best knowledge of authors, LoRa technology is yet to be introduced in an underground mine for location tracking purpose of miners.

### State of the art of location estimation using VLC system

Due to the advancement of LED technology, VLC system is seeking huge attention from the researchers. In [46] VLC propagation channel is being considered to establish communication link for two cases: miner-to miner communication and infrastructure-to-miner communication. The work shows that if LOS is maintained, then accurate channel model can be developed with first order reflections. Along with the channel model, authors also investigated illumination criteria for two parts of mine: working face and mining roadway and from the experiment it can be said that working face needs more illumination than roadways.

The work in [47] examined channel model characteristics. A path loss channel model was introduced based on recursive model and bimodial Gaussian distribution was used to study the shadowing effect of VLC system in underground mine. The path loss model was developed to show distance dependence and linear relationship in log domain. The authors considered both the LOS and NLOS scenario.

VLC system was deployed in a coal mine workface in [48]. A mathematical model was developed to theoretically study the energy coupling of mining machineries and effect of coal dust in propagation of optical signal transmission. As a result optimal position for the optical transmitter was determined so that coupled energy can be maximized.

Another application of VLC system is mentioned in [49] where three dimensional trilateration was introduced for location estimation inside the mine. The proposed algorithm was based on average localization error and the average error was reduced to 3.5 cm by the group.

The work in [50] elaborated channel impulse response (CIR) for a VLC system to ensure safe and reliable mining operation considering LOS communication. Optical channel model was proposed and effect of dust and shadowing was tested. Bit error rate was degraded by the shadowing effect which disrupted the stability of the communication link.

Theoretical study of optical transmitter model, optical receiver model, channel model and propagation model is given in [51]. Also channel characteristics like; gain, impulse response and reception power and illumination property was studied. As a conclusion of all the studies, it can be said that by maintaining LOS, VLC system can be successfully implemented in underground mine. Table 6 presents work done on VLC system in the past decade. It also includes the contribution of the present work.

**Table 6:** Literature comparison of last 10 years work on VLC system and presented work

Paper/year	Application area	Adopted technique	Purpose of the work	Obtained result
[52]/2011	Indoor Environment	Positioning technique	Illumination and positioning	System is able to detect position of the receiver (image sensors) with the accuracy of 10 cm
[29]/2012	Indoor environment	AOA technique	Indoor location estimation of mobile station	To yield better AOA estimation and location estimation, more number of PDs is to be deployed. A truncated weighting scheme is proposed
[32]/2013	Indoor environment	TOA technique	Indoor positioning estimation	Geometry of the room, frequency and power of the transmitted signal and properties of the optical components decides the accuracy of the location estimation algorithm
[31]/2014	Indoor environment	RSS technique	Indoor positioning estimation	With 95% confidence, achieved accuracy upto 5.9 m in estimating location
[27]/2014	Indoor environment, inside a building	Bayesian signal model	Self-localization of mobile devices	Synchronization of transmitter can be ignored. The system is robust against obstructions of LOS. And also excellent performance with minimum complexity is achieved
[34]/2015	Indoor environment	AOAtechnique	Estimation of AOA parameters for three dimensional visible light positioning	From the proposed AOA algorithm it is possible to measure all the AOA parameters from visible light signal at the receiver
[48]/2015	Underground Mine (Work face)	Ray transmission method and Mie Scattering	Effect of coal dust on optical signal degradation	Estimating sheltering effect of metal pillar, emitter position and receiver signal energy and power estimation can be determined
[38]/2016	Indoor environment	Positioning technique	Analysing of the performance of the visible light positioning system consisting of aperture based receiver and measurements of received signal strength	Compact directional receiver and limited number of LEDs can be used for positioning
[46]/2017	Underground Mine (Work face + roadway)	Recursive method	Illumination and VLC channel characteristics	To yield better performance of the system channel, LOS and first order reflection need to consider
[47]/2018	Underground Mine (Work face + roadway)	Recursive model	Path Loss channel model for VLC system	Path loss expression is obtained which is dependent on transmitter and receiver distance. RMS delay and mine scattering is analysed
[49]/2019	Underground Mine (Work face + roadway)	Three-dimensional trilateration VLC localization scheme	Localization in underground Mining	Localization estimation error of less than (16.4 cm)
[51]/2019	Underground Mine (Work face + roadway)	Monte Carlo method	Illumination, channel impulse response and power distribution in reception	Received power is directly proportional to the distance between optical elements
[50]/2020	Underground Mine (Work face + roadway)	Intensity modulation and direct detection technique	Communication Facility, Illumination	Observed system performance in presence of mine dust particles and shadowing
Presented Work	Underground Mine (Work face + roadway)	RSS Technique	Estimation of Signal-To-Noise Ratio with VLC system	Interference level is reduced by adopting zone division technique. And SNR show promising result at the centre of each zone
		Mathematical Model	Estimation location of miners	Algorithm proposed

## Proposed system model

Location estimation of miners in underground mine environment is challenging but with the advent of technologies it is possible to calculate the approximate location of miners. In this paper, VLC system is used and with the knowledge of RSS, an algorithm is proposed that will help to calculate the approximate location of a miner working underground. The main aim of the presented work is to gather information regarding location of miners.

### System development

**Fig. 1:**
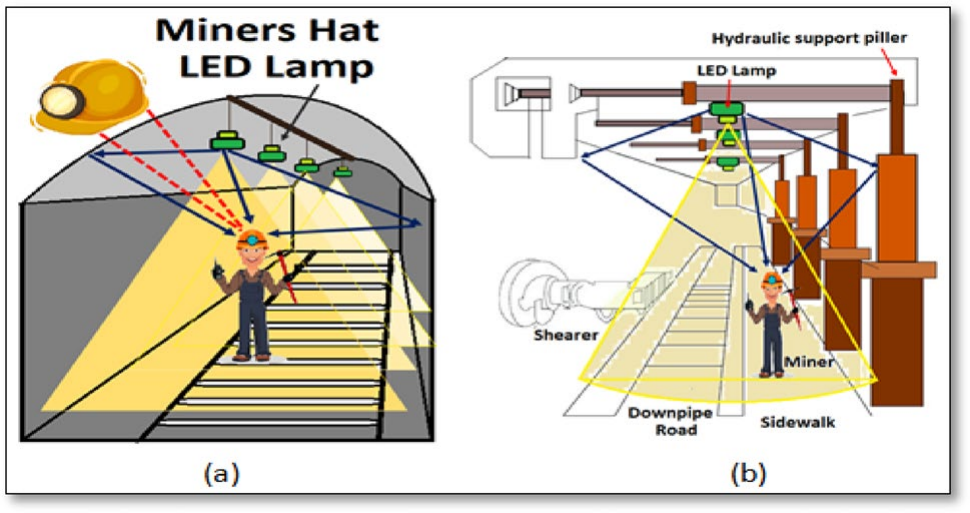
System model: **a** horseshoe mining roadway, **b** mining workface

For implementing VLC system inside a coal mine, LEDs are to be placed at the roof of the mine that will act as transmitter. Safety helmet of the miners will be equipped with receiver unit. The helmets will be fitted with PDs, signal processing unit and transceiver unit. Optical receiver placed on the miner’s safety helmet will receive the signal. Optical transmitter and receiver will communicate when LOS is established. Novelty of the work lies in sectorization of the whole mine area. Optical components of VLC system will work better when LOS is established. Dividing mine area into smaller zones will help reduce intercellular interference. Mine area can be identified as roadway and workface. Figure 1a, b show structure of roadway and workface of a mine respectively.

Roadway is a tunnel through which minerals reach the surface and workface is a surface where excavation operation is carried out. Workface is surrounded by thick walls, whereas, roadway is a long open tunnel. In the workface area, zones will be separated from each other by means of long walls and pillars present in the mine. But in the roadway, zones can be identified by means of field of view (FOV) of the LEDs. FOV of each LED is the virtual partition in roadway area, and these are referred to as zones here. Partitions through LEDs are not physical partition.

LEDs are to be placed at the ceiling of roadway and workface, as shown in Fig. 1. Every miner will wear a safety helmet fitted with optical receiver unit. In roadway area shown in Fig. 1a, when one miner arrives near a LED, it will enter the FOV zone of that particular LED. LEDs in this area will be placed at a pre-determined distance from each other, so that no two adjacent zones will overlap with each other. Aim is to minimize the intercellular interference. In workface area, upon arriving in a zone, shown in Fig. 1b, miner’s receiver unit will receive signal from LED of that zone. The transceiver unit of the optical receiver will transmit necessary information to the above the surface control room. Based on the information, location of that miner can be estimated.

### SNR calculation for VLC system

The proposed algorithm is based on RSS. MATLAB simulation is done to observe the SNR performance for VLC system. For test purpose a small area has been selected, where the LED is placed at the point near the ceiling and receiver is placed on the ground. Other parameters that are considered are listed in Table 7.

**Table 7:** Specifications for simulation

Width of the room	10 m
Length of the room	5 m
Height between transmitter (LED) and receiver (PD)	10 m
Responsivity of PD	0.55 A/W
Transmitter FOV	70 deg
Receiver FOV	90 deg

### Proposed algorithm

This work proposes an algorithm to calculate the location of the miner in underground mine. The algorithm works with the knowledge of the SNR performance on VLC system. Proposed algorithm uses Intensity modulation/direct detection (IM/DD) technique for modulation and demodulation of the signal. Further, for ease of calculation it can be assumed that cos (φ) = cos (θ) [this is done with the assumption that PD is parallel to roof].

Some parameters that are being measured at the design level are listed below:

- Physical area of PD within the receiver → A_t_,
- Gain of the optical filter → T_s_(θ),
- FOV of PD → θ_c_,
- Refractive index → n,
- Height of the LED from ground → h_i_,
- Height of the PD → h_p_.

The steps of the proposed algorithm are as follows:
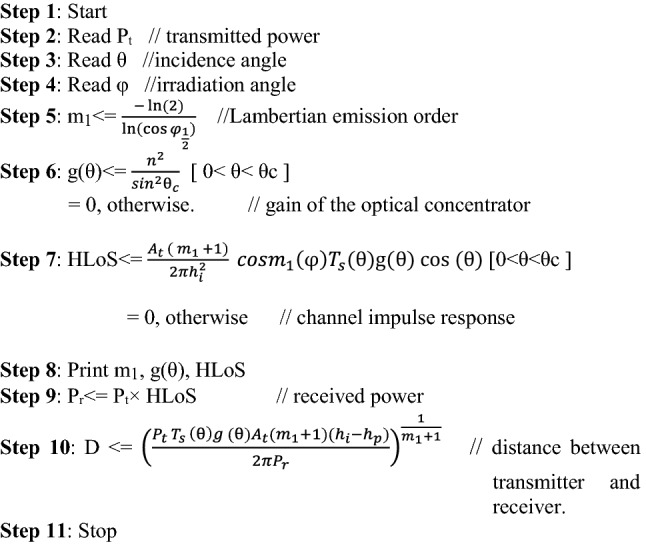


### Data transmission

**Fig. 2:**
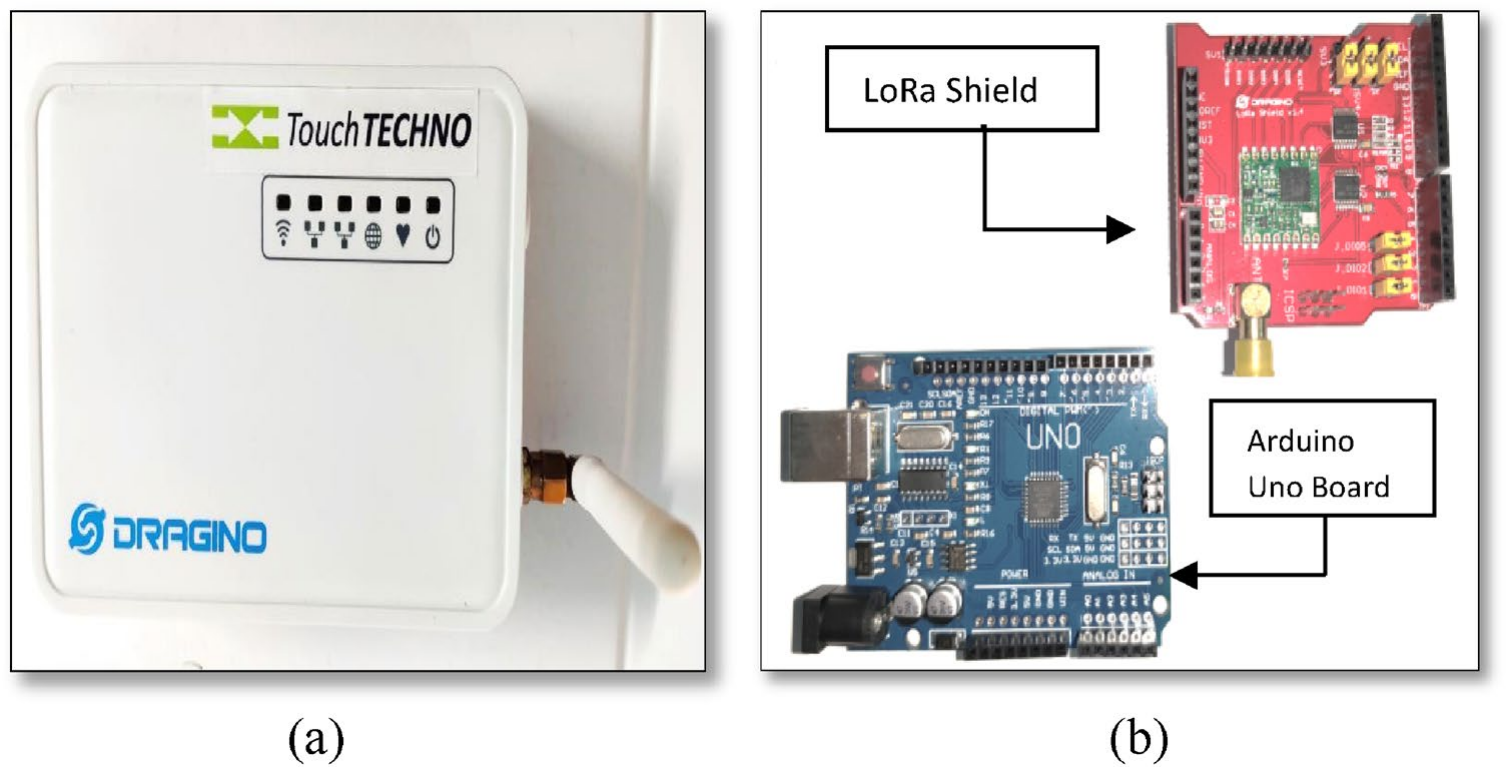
**a** LG01-N LoRa Gateway for 868 MHz frequency; **b**: LoRa shield with Arduino Uno Board

The LoRa transceiver, also known as LoRa shield is placed in the receiver unit of VLC system. This transceiver unit when attached with any sensor will be able to transmit the collected data to server. On the safety helmet of the miners, PD will act as a sensor. LoRa shield will be connected with the sensor and together will act as an end node. End node will then communicate with the gateway using unlicensed 868 MHz frequency (in India). Gateway is responsible to collect the data from end node and to store the data to a server through network server; the things network (TTN). Figure 2a, b depict one LG01-N LoRa gateway and LoRa shield along with one microcontroller respectively.

Range test was performed under three different scenarios; indoor, outdoor and underground tunnel, using the gateway and 50 dB antenna. Due to Covid19 situation, the test was initially carried out in an underground tunnel. The same test was conducted in an underground mine at Pandaveswar Colliery at a later date.

## Results and discussion

Simulation has been performed using MATLAB software with the parameters declared in Table 7. In this section, two different results has been discussed,

(i) SNR performance of VLC system and,
(ii) Range test results with LoRa technology and comparing it with established technologies like Wi-Fi and ZigBee.

### SNR performance of VLC system

**Fig. 3:**
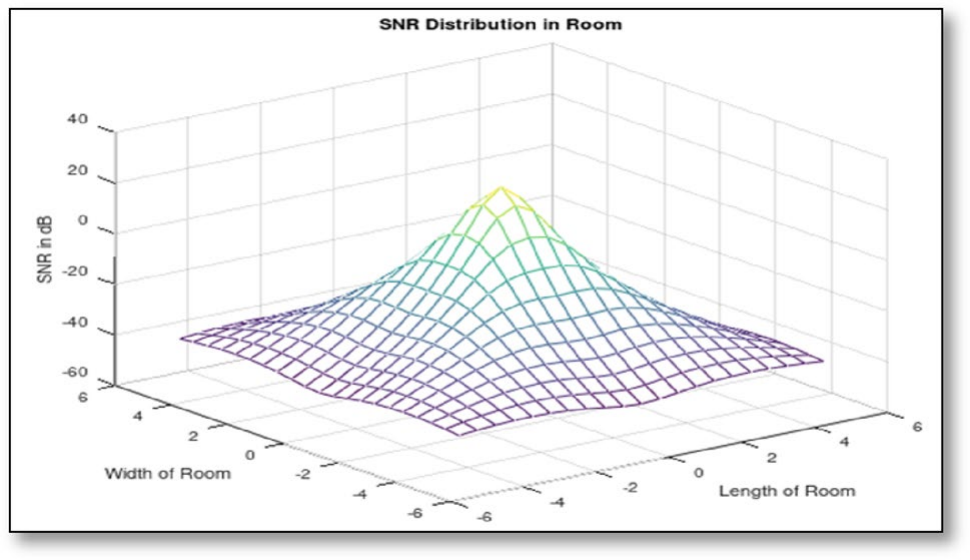
SNR performance of a VLC system

**Fig. 4:**
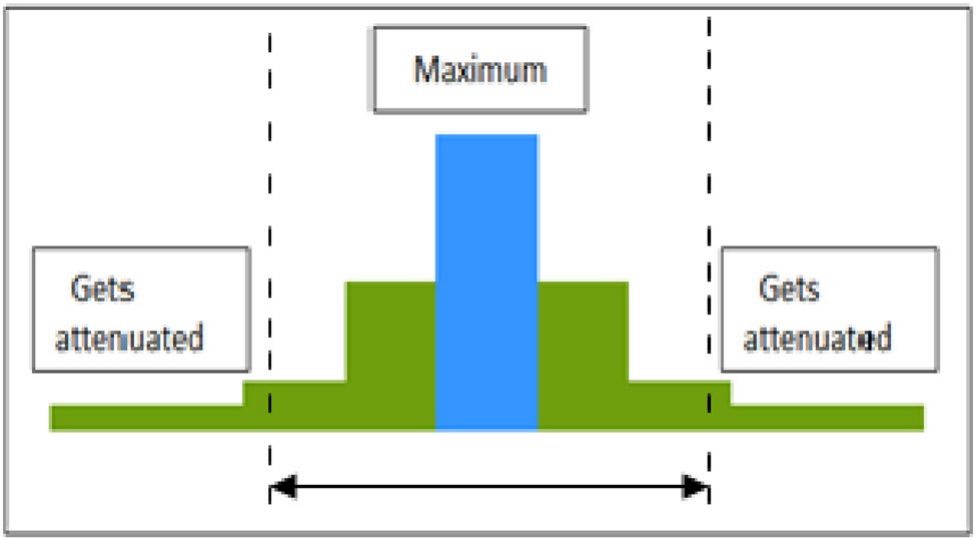
Received power distribution at receiver unit

The SNR performance has been calculated using MATLAB simulation. Result of the simulation is presented in Fig. 3. The result shows that SNR is strong at the center of a zone. A high value of SNR implies noise can be eliminated in order to receive satisfactory signal strength at the receiver. For justification of the obtained SNR characteristics, received power distribution graph is presented in Fig. 4. While running the algorithm, it is very important to consider the strongest signal for better accuracy. Dashed line in Fig. 4 represents the allowable received power that can be considered for location estimation calculation. With the strongest signal, calculation of other parameters mentioned in the algorithm will be performed with high accuracy.

From both the graphs, it is evident that SNR value decreases at the edges of the defined zone. SNR value could decrease, if strong interference is experienced by the receiver. But with the novel zone division method, this interference can be almost nullified. In the proposed method, decrease in SNR only means the miner moving away from the center point to the edge of the zone. Once the miner leaves that zone he will be latched with adjacent zone's LED. Thus signal overlapping problem is also reduced in this method. This concept will help in locating the miner's position accurately with respect to the transmitting LED.

### Range test for data transmission mechanism

The signal transmission range needs to be determined before deploying any communication system in underground mine. Existing technologies for location tracking, like WLAN and WPAN fail to provide long range inside mine. This is conclusively proved in an earlier work of this group [6].

Range test result of WPAN and WLAN from paper [6] along with the range test result with LoRa technology is presented in Table 8.

**Table 8:** Comparison of transmission range from different networks for different environments

Network	Outdoor environment (approximately) [m]	Indoor environment (approximately) [m]	Mine environment (approximately)
WPAN	35	18	13 m
WLAN	95	28	17 m
LoRa	130	40	29 m (underground tunnel)
			28.82 m (underground mine)

From Table 8 it can be seen that the transmission range obtained using LoRa technology is much higher than the existing technologies. Due to COVID-19 situation, field visit was not possible during the initial phase of the work so the authors carried the experimentation in an underground tunnel. The obtained result using 50 dB LoRa gateway is much better than that using WLAN or WPAN. Later LoRa range test was done using the same gateway at the underground Pandaveswar Colliery and the obtained range is almost same as that obtained in the underground tunnel. Thus it can be said that result obtained from proposed system in underground tunnel will also be true in underground mine. Inside the mine, increased communication range can also be achieved by using high power antenna and multiple industrial gateways.

## Conclusions

The work presented here offers a potential solution to improve the working condition inside an underground mine. Incorporating VLC and LoRa technology enables IOT facility inside a mine. The paper also proposes an algorithm to measure the distance between optical transmitter and receiver. The presented simulation results demonstrate that the RSS is strongest at the center of each zone. This paper also exhibits that range test experimentation with LoRa technology, in underground tunnel gives almost similar results as that in underground mine. Experimentally it has been shown that the communication range inside a coal mine with WPAN and WLAN is 13 m and 17 m respectively. But LoRa technology with gateway of 50 dB antenna offers an improved signal transmission range of 28.82 m.
